# Forward and Backward Unidirectional Scattering by the Core-Shell Nanocube Dimer with Balanced Gain and Loss

**DOI:** 10.3390/nano10081440

**Published:** 2020-07-23

**Authors:** Jingwei Lv, Xiaoming Zhang, Xuntao Yu, Haiwei Mu, Qiang Liu, Chao Liu, Tao Sun, Paul K. Chu

**Affiliations:** 1 School of Physics and Electronic Engineering, Northeast Petroleum University, Daqing 163318, China; lvjingwei2009123@126.com (J.L.); mhwmzh@163.com (H.M.); nepulq@126.com (Q.L.); 2 College of Physics Science and Engineering Technology, Yichun University, Yichun 336000, China; zhangxm8555@163.com; 3 School of Earth Sciences, Northeast Petroleum University, Daqing 163318, China; yxt0407@163.com; 4 Media Lab, Massachusetts Institute of Technology, Cambridge, MA 02139, USA; taosun@hotmail.com.hk; 5 Department of Physics, City University of Hong Kong, Hong Kong 999077, China; paul.chu@cityu.edu.hk; 6 Department of Materials Science and Engineering, City University of Hong Kong, Hong Kong 999077, China; 7 Department of Biomedical Engineering, City University of Hong Kong, Hong Kong 999077, China

**Keywords:** backscattering, forward scattering, nanoantenna, spectra

## Abstract

An optical nanoantenna consisting of a Au-dielectric core-shell nanocube dimer with switchable directionality was designed and described. Our theoretical model and numerical simulation showed that switching between forward and backward directions can be achieved with balanced gain and loss, using a single element by changing the coefficient *κ* in the core, which can be defined by the relative phase of the polarizability. The optical response indicated a remarkable dependence on the coefficient *κ* in the core as well as frequency. The location of the electric field enhancement was specified by the different coefficient *κ* and, furthermore, the chained optical nanoantenna and coupled electric dipole emitted to the optical nanoantenna played significant roles in unidirectional scattering. This simple method to calculate the feasibility of unidirectional and switchable scattering provides an effective strategy to explore the functionalities of nanophotonic devices.

## 1. Introduction

Metallic nanostructures featuring a multitude of localized and propagating surface plasmon resonances have attracted significant interest due to their ability to concentrate and manipulate light at the subwavelength scale [1,2]. Their extraordinary properties have been widely exploited in a broad range of optical applications, including solar cells [3,4], ultrasensitive sensors [5], photovoltaic devices [6,7], and so on. However, relatively high intrinsic ohmic loss and local heating in the metallic nanostructures have impeded wider acceptance [8,9] and therefore, there has been increasing interest in new materials such as semiconductor crystals and dye molecules in order to produce plasmonic nanostructures with low dissipative losses [10,11].

Metallic nanostructures with engineered optical properties constitute a new platform to tailor light–matter interactions on active materials [12]. Optical nanostructures with hybrid active–passive geometries rely on the balance between gain and loss [13,14] and active–passive nanostructures shed light on important non-Hermitian quantum mechanics. In the optics community, novel applications based on effects such as coherent perfect absorption [15], non-linear switching [16], and asymmetric transmission [17] have been proposed. Although many papers have focused on guided wave systems and lattices, the scattering arrangements in two- and three-dimensional geometries attainable with active–passive nanostructures have been less explored [18,19,20,21,22]. Large flexibility is particularly attractive with respect to the design and optimization of coupling between different materials and hence, it is important to investigate the unique and unidirectional optical properties of active–passive nanostructures and the scattering response of electromagnetic waves.

In this article, an optical nanoantenna, consisting of a core-shell nanocube dimer and encapsulated with an outer Au shell doped with active and passive nanocubes, was designed for balanced gain and loss and evaluated by the finite element method (FEM). Gold nanoparticles (AuNPs) provide an excellent platform for biological and material applications due to their unique physical and chemical properties. AuNPs are chemically inert and oxidation-free and also show high biocompatibility, when compared with silver, aluminum or other nanomaterials [23,24,25]. The dielectric core, by controlling the imaginary part of the refractive index *κ* in loss and gain parts, can be made either from dye molecules, semiconductor nanoparticles with sizes of a few nanometers, or from rare earth ions [26,27,28]. The effects of the imaginary part of the refractive index *κ* in the core and frequency were studied to confirm and understand the unidirectional line shape and tunability of the system. The far-field radiation patterns of scattered light can be switched from primarily forward scattering to backward scattering by simply varying the coefficient *κ* in the core, making them interesting components of optical devices used to actively control light at the nanoscale. The core-shell nanocube dimer with balanced gain and loss can act as a directional nanoantenna, both in transmitter and receiver modes [29]. Furthermore, the unidirectional scattering properties of the core-shell nanocube dimer with balanced gain and loss arranged in a chain were determined and directional emission from the electric dipole emitter was demonstrated.

## 2. Theory

**Figure 1:**
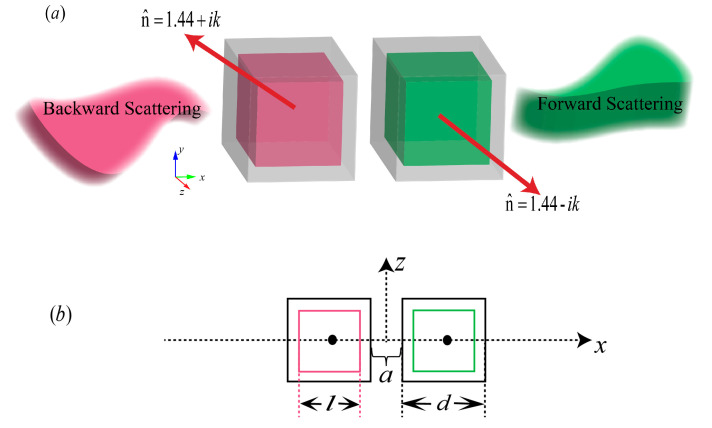
Schematic of the core-shell nanocube dimer with balanced gain and loss. (**a**) three-dimensional diagram (**b**) *x-z* plane graph.

We considered the optical nanoantenna composed of a core-shell nanocube dimer as shown in Figure 1. It is composed of an inner dielectric core coated with an outer cubic Au nanobox in Figure 1a. A model system was considered in which the coefficient *κ* was introduced to the core medium to describe the phenomenological response of the incident light to the gain and loss of the materials. The dielectric core has a refractive index *n* ± *iκ*, with one corresponding to loss media (+*κ*) in the left cube and the other corresponding to gain media (−*κ*) in the right cube. We considered n = 1.44, corresponding to the refractive index of SiO_2_, in the frequency range of interest. The coefficient *κ* was always the same for both cores to satisfy the balanced gain and loss condition. The optical constant of gold was obtained from Palik’s handbook [30]. The *x-z* plane graph is shown in Figure 1b. The metal and dielectric edge lengths, *d* and *l*, were set as 50 nm and 40 nm, respectively. The face-to-face distance was = 20 nm. In the simulation, the nanostructure was assumed to be freestanding in air (the dielectric constant *ε_d_* = 1), and a plane wave impinged onto the hybrid core-shell nanocube dimer along the *x* direction with $E_{z}$ polarization. The scattering properties were investigated by the finite element method with COMSOL Multiphysics [31]. Following geometrical model definition, the nanostructure was meshed. Tetrahedral meshing was used in the frequency-domain simulations with automatic mesh refinement to study the scattering pattern. The simulation domain was finely meshed with a minimum mesh of 1 nm and maximum mesh of 5 nm over the volume of the core-shell nanocube dimer. In all the simulations, the core-shell nanocube dimer was surrounded by a spherical shell of perfectly matched layer (PML) and the maximum meshing size was chosen to be a sixth of the wavelength, and a total mesh number of 44.270 was used over the whole system. The average element quality was 0.75 such that the convergence of the results and the accuracy of the computed quantities were ensured.

## 3. Results and Discussion

**Figure 2:**
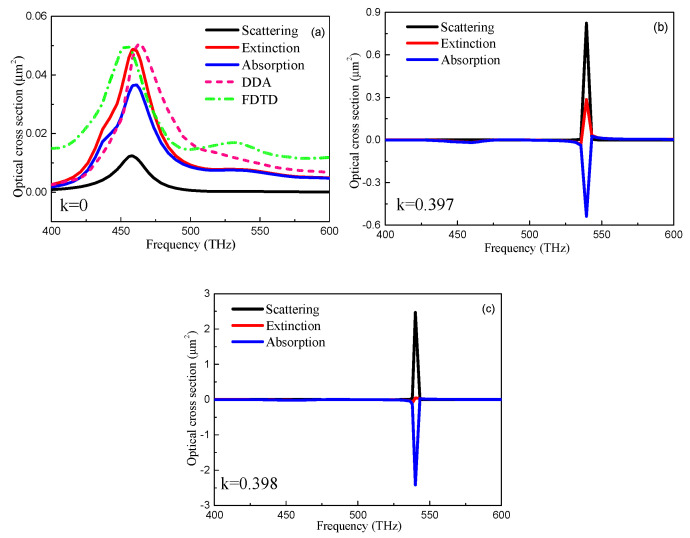
Calculated optical cross-section spectra of the core-shell nanocube dimer with a different coefficient *κ* in the core. (**a**) *κ* = 0 (**b**) *κ* = 0.397 (**c**) *κ* = 0.398.

In order to offer a better understanding of the optical properties, we show in Figure 2 the calculated spectra of absorption (*C*_abs_), scattering (*C*_sca_), and extinction (*C*_ext_) cross section of the core-shell nanocube dimer with the different gain and loss coefficient *κ*. C_sca_ is defined as the optical cross section for both forward and backward scattering. As expected, when *κ* = 0, there were two plasmon peaks at $\upsilon_{1}=460\;\mathrm{THz}$ and $\upsilon_{2}=540\;\mathrm{THz}$ and the *C*_ext_ values were about 0.05 μm^2^and 0.008 μm^2^. The result of the extinction cross section was in good agreement with numerical calculations by the Discrete Dipole Approximation (DDA) and Finite difference time domain (FDTD) simulations. It is noted that there only existed a small deviation of frequency for the optical cross section between different numerical results. The low quality factor of plasmonic peaks can be primarily ascribed to strong dissipation of conduction electrons in the Au nanobox. Additionally, C_abs_ was about three times that of C_sca_, indicating intense absorption by the core-shell nanocube dimer at the coefficient *κ* = 0 in Figure 2a. As the coefficient *κ* was increased to 0.397, scattering of nanocube dimer at $\upsilon_{2}=540\;\mathrm{THz}$ increased to about 0.79 μm^2^ and *C*_abs_ became −0.53 μm^2^ in Figure 2b. The negative *C*_abs_ can be interpreted, as that scattered radiation was compensated by the energy amplification from the gain materials. *C*_ext_ also increased remarkably due to the compromise between the scattering loss and amplification gain. As *κ* was increased to the critical value of 0.398, a significant resonance peak was observed at $\upsilon_{2}=540\;\mathrm{THz}$ as shown in Figure 2c. *C*_sca_ increased to a 2.5 μm^2^ which is about 250 times that of the passive core-shell nanocube dimer and *C*_abs_ decreased to a minimum of about −2.5 μm^2^; in this case, the gain overcame the loss and C_abs_ reached the minimum value. The relatively sharp peak compensated for *C*_sca_ to yield zero extinction. This verifies the dynamic balance between light amplification and dissipation in the nanostructure and indicates the lasing threshold [32,33].

**Figure 3:**
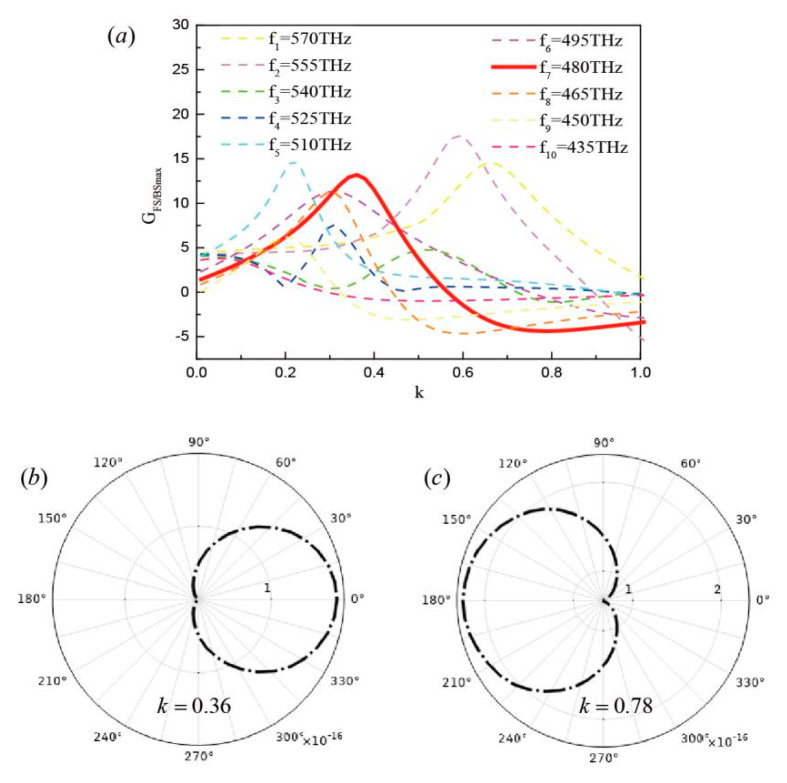
(**a**) Far-field forward-to-backward directionality $G_{FS/BS_{max}}$ for different frequencies. (**b**) Scattering pattern for *κ* = 0.36. (**c**) Scattering pattern for *κ* = 0.78.

According to the discussion above, the core-shell nanocube dimer with balanced gain and loss at $\upsilon_{2}=540\;\mathrm{THz}$ was chosen to study unidirectional scattering. The far-field scattering patterns for the coefficient *κ* in the core as a function of frequency around $\upsilon_{2}=540\;\mathrm{THz}$ are shown in Figure 3a. The far-field forward-to-backward directionality of the core-shell nanocube dimer with balanced gain and loss in dB can be obtained by $G_{FS/BS_{max}}=10\log_{10}(S_{F}/S_{B})$, where $S_{F}$ is the amplitude of the power radiated in the forward (x>0) and backward (x<0) directions [34,35]. The direction of the scattered wave changes as the coefficient *κ* in the core is varied. For example, by changing the frequency between $f_{10}=435\;\mathrm{THz}$ and $f_{1}=570\;\mathrm{THz}$, the directionality $G_{FS/BS_{max}}$ was altered. In particular, the bold curve at the frequency of $f_{7}=480\;\mathrm{THz}$ exhibited pronounced directionality towards the opposite direction. The coefficient *κ* = 0.36 in the forward direction was about 14 dB stronger than in the backward direction and that of *κ* = 0.78 was directed toward the backward direction and about 5 dB stronger than in the forward direction. In general, the bold curve at the frequency of $f_{7}=480\;\mathrm{THz}$ satisfied the largest change in directionality and a change in magnitude from negative to positive. Figure 3b,c further shows that the scattering direction of the antenna at a fixed frequency can be reversed by adjusting *κ*. This shows switchable and highly directional radiation for different coefficient *κ* values in the core originating from constructive and destructive interference in the backward and forward radiation [36]. This was set as the default frequency in our calculation and the directionality can be tuned by the frequency. To clarify the influence of the geometry on the far-field scattering patterns for the coefficient *κ* in the core, a systematic investigation was performed by varying three parameters *d*, *l*, *a*. The corresponding models were optimized, and the calculated spectra show the different scattering effects for the directions (see Supplementary Figure S1). The directionality $G_{FS/BS_{max}}$ reached the maximum and a high directivity of scattering could be achieved when *d*, *l*, *a* were 50 nm, 40 nm, and 20 nm, respectively. Therefore, the directionality $G_{FS/BS_{max}}$ is described based on geometric parameter optimization to realize superior directionality towards the opposite direction.

**Figure 4:**
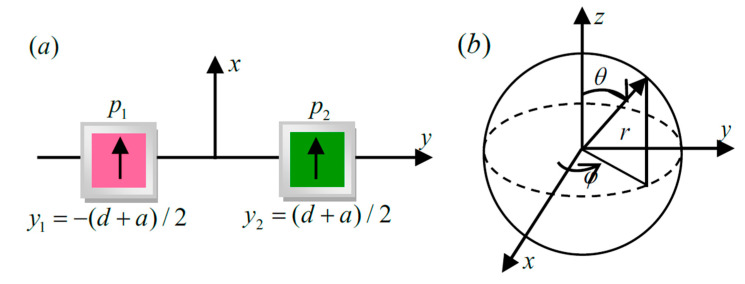
(**a**) Schematic of the coupled dipole mode and (**b**) Spherical coordinates for the core-shell nanocube dimer with balanced gain and loss.

To benchmark our numerical calculation, we calculated the power difference ΔP with an analytic dipole model for the far-field directionality of the core-shell nanocube dimer with balanced gain and loss [37]. The two emitting dipoles with moments defined as $p_{1}$ and $p_{2}$, were positioned on the *-x* axis and *+x* axis, respectively. Their *y*-coordinates were $x_{1}=-(d+a)/2$ and $x_{2}=(d+a)/2$, respectively (see Figure 4a). In the far-field limit (r ⪢ λ),

(1) $$\begin{array}{l} \left|r-x_{j}\right|-r=\sqrt{{\left(r-x_{j}\right)}^{2}+y^{2}+z^{2}}-r\\ \approx r(\sqrt{1-2xx_{j}/r^{2}}-1)\\ \approx -x_{j}(x/r)\approx -x_{j}\sin(\theta )\cos(\varphi ) \end{array}$$

The electric and magnetic fields excited by the core-shell nanocube dimer in the far-field are represented as $p_{1}$ (*j* = 1) and $p_{2}$ (*j* = 2). Therefore, the electric field vector $E_{j}$ and magnetic vector $H_{j}$ produced in the far-field can be written as follows:

(2) $$\begin{array}{l} E_{j}(r,\theta ,\varphi )=\frac{b^{2}}{4\pi \varepsilon_{0}}e^{ib\left|r-x_{j}\right|}[(e_{r}\times p_{j})\times e_{r}]\\ =\frac{b^{2}}{4\pi r\varepsilon_{0}}e^{ibr}e^{-bx_{j}\sin(\theta )\cos(\varphi )}p_{j}\sin(\theta )(-e_{\theta }) \end{array}$$

(3) $$H_{j}(r,\theta ,\varphi )=\frac{b\omega }{4\pi r\varepsilon_{0}}e^{ibr}e^{-ibx_{j}sin(\theta )cos(\varphi )}p_{j}\sin(\theta )(-e_{\varphi })$$

where $e_{r}$, $e_{\theta }$ and $e_{\varphi }$ are the unit vectors of the spherical basis (Figure 4b). *b* is the propagation constant in the background medium (1/m). $\varepsilon_{0}$ is the dielectric constant of vacuum $8.85\times 10^{-12}(F/m)$. *r* is the distance from the system structure to the observation point r=1 m. The frequency was set as f=480 THz, which was obtained by Figure 3. Scattering by the core-shell nanocube dimer in the far-field can be obtained by the time-averaged Poynting vector of the sum of these fields [38]:

(4) P(r,θ,φ)=12Re[(E1+E2)*×(H1+H2)]=ω3b32π2ε0c2r2(p1*eibre−ibx1sin(θ)cos(φ)+p2*eibre−ibx2sin(θ)cos(φ))×(p1eibre−ibx1sin(θ)cos(φ)+p2eibre−ibx2sin(θ)cos(φ))×sin2(θ)er=ω3b32π2ε0c2r2[|p1|2+|p2|2]+2Re(p1p2*eib(d+a)sin(θ)cos(φ))sin2(θ)er

To study unidirectional scattering in the far-field, the sum of the Poynting vector in one direction and in the opposite direction was calculated:

(5) ΔP(r,θ,φ)=P(r,θ,φ)+P(r,π−θ,φ+π)=ω3b16π2ε0c2r2Re{2ip1p2*sin[b(d+a)sin(θ)cos(φ)]}sin2(θ)er

To elucidate the physical mechanism for the far-field directionality, the power difference ΔP was described as follows:

(6) $$\Delta P=P_{1}-P_{2}$$

**Figure 5:**
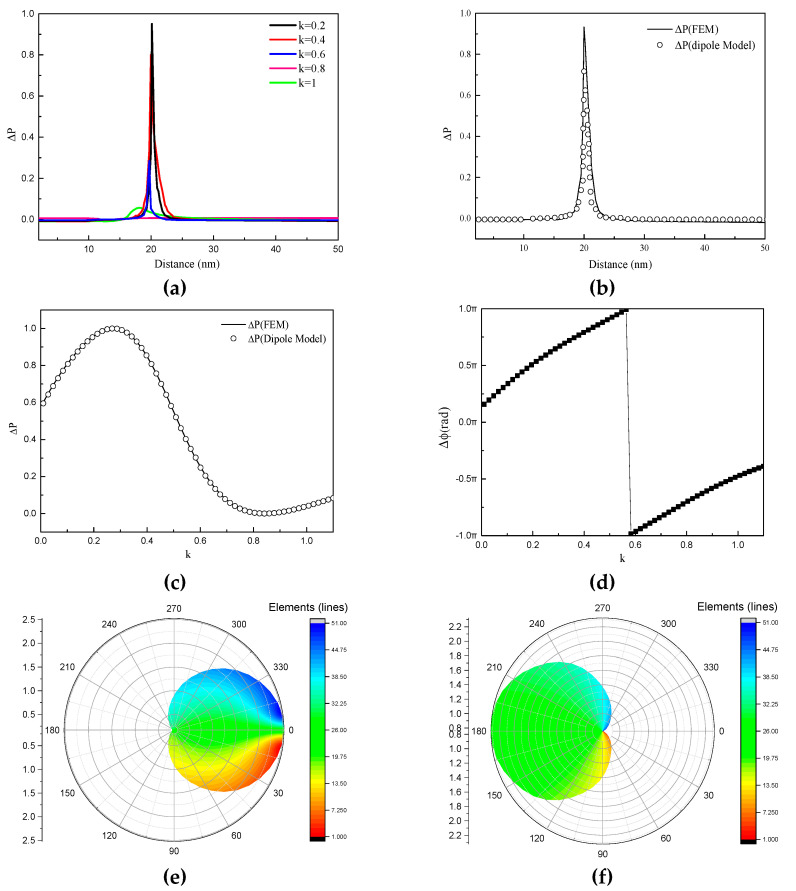
(**a**) Power difference ΔP spectrum with face-to-face distance of the core-shell nanocube dimer (**b**) Power difference ΔP spectrum with different face-to-face distance obtained by the analytic expression and finite element method (FEM) (**c**) Power difference ΔP spectrum with different *κ* obtained by the analytic expression and FEM (**d**) Phase difference between the electric dipoles (EDs), (**e**) Far-field distributions at *κ* = 0.268, and (**f**) Far-field distributions at *κ* = 0.84.

To estimate how much light was scattered by the core-shell nanocube dimer with balanced gain and loss, we defined $P_{1}$ as the far field power emitted along the +*x* axis and $P_{2}$ the far-field power emitted along the -*x* axis. As can be seen in Equation (6), a significant enhancement in the forward (backward) direction was achieved in conjunction with suppression in the backward (forward) direction. The power difference ΔP spectrum as a function of the face-to-face distance of the core-shell nanocube dimer is calculated in Figure 5a. It can be seen that the power difference ΔP is very sensitive to the variation of the face-to-face distance of core-shell nanocube dimer. Moreover, the maximum power difference ΔP was approximately 0.95 at *a* = 20 nm, and ΔP changed dramatically in the range of *a* = 15–25 nm as *κ* increased from 0.2 to 1. Therefore, the optimized parameter of face-to-face distance *a* = 20 nm was set to calculate the power difference ΔP.

The electric dipole moment can be written as $p_{1}=\left|p_{1}\right|e^{i\phi_{1}}$ and $p_{2}=\left|p_{2}\right|e^{i\phi_{2}}$, where $\phi_{1}$ and $\phi_{2}$ are the phases of the electric dipole moment $p_{1}$ and $p_{2}$. Assuming that the relative phase is $\phi =\phi_{1}-\phi_{2}$:

(7) ΔP(r,θ,φ)=ω3b16π2ε0c2r2×Re{2|p1||p2|sin(ϕ)sin[b(d+a)sin(θ)cos(φ)]}sin2(θ)er

According to Equation (7), when ϕ=tπ(t∈N), ΔP=0 for any dipolar amplitudes $\left|p_{1}\right|$ and $\left|p_{2}\right|$. For θ=π/2 and φ=0 is the position of the electric dipoles along the *x* axis, we have:

(8) ΔP=ω3b16π2ε0c2r2Re{2|p1||p2|sin(ϕ)sin[b(d+a)]}x

Equation (8) provides the general guideline to calculate the power difference ΔP $\left|p_{1}\right|$, $\left|p_{2}\right|$ and ϕ are varied with face-to face distance *a* and *κ*, which means the magnitude and phase of the electric dipole moment change when *a* and *κ* change. As shown in Figure 5a, the peak of the ΔP spectrum shifts to a shorter wavelength when the coefficient *κ* is increased. The face-to face distance dependence of the power difference ΔP for k=0.2 as shown in Figure 5b, is in agreement with the simulation results. The power difference spectrum of the core-shell nanocube dimer was derived by the FEM as a function of coefficient *κ* in the core as shown in Figure 5c which revealed a peak and valley at k=0.268 and k=0.84 similar to a sine wave.

As shown in Figure 5c, the dissimilarity was obtained at about 0.072 by using a very simple Kolmogorov–Smirnov test to quantify the difference between the analytic expression and FEM, the results (circles) superimposed onto the FEM calculation (solid line) indicate excellent agreement between the theory and the simulation. The relative phase of the two electric dipoles were approximately in the opposite phase for k=0.268, Δϕ=π/2 and k=0.84, Δϕ=−π/2 (Figure 5d). In this case, directional scattering could be achieved and the far-field scattering pattern of particles was concentrated in the direction of the incident wave. Moreover, as shown in Figure 5e the radiated far-field was directed towards the forward direction for k=0.268. In contrast, as shown in Figure 5f the radiated far-field was directed towards the backward direction. The far-field distributions can be explained with the two-dipole model provided by Figure 5c. When k=0.268, and thus ΔP>0, this means that forward scattering is significantly enhanced while the backward scattering is considerably suppressed. One can see that ΔP<0 when k=0.84, the suppression of forward scattering and enhancement of the backward scattering was observed. Therefore, emission of the local light source can be directed and switched between two opposite directions based on the coefficient *κ* of the core-shell nanocube dimer with balanced gain and loss. We propose to use these nanoantennas with balanced gain and loss as optical emitters or receivers, whose application would be useful in the direct optical links of future nanophotonic radio frequency (RF) circuits [39].

**Figure 6:**
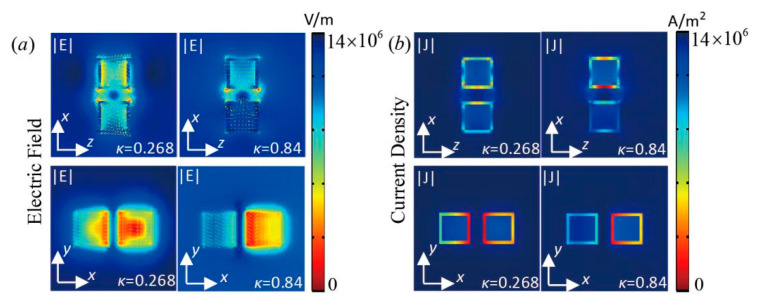
(**a**) Electric field distribution and (**b**) Current density of the core-shell nanocube dimer for *κ* = 0.268 and *κ* = 0.84.

To obtain more information about the optical properties of the core-shell nanocube dimer, the electric field distribution and current density at *κ* = 0.268 and *κ* = 0.84 are presented in Figure 6. The near-field distributions were determined by electric dipolar excitation of the core-shell nanocube dimer. For the *x-z* plane, the electric field hotspot occurred naturally at the corner region and the inner core (Figure 6a). The electric field magnitude on the two sides of the core-shell nanocube dimer for the *x-y* plane was at *κ* = 0.268 and the field was dispersed on the entire surface of the gain part at *κ* = 0.84. There was strong field localization and the electric field enhancement stimulated the optical response in the nanoantenna. At the same time, a large portion of the electromagnetic energy was localized at the outer cubic Au nanobox (Figure 6b) and the peak and valley at k=0.268 and k=0.84 were derived from the interaction of the electric dipoles according to the current density distributions.

**Figure 7:**
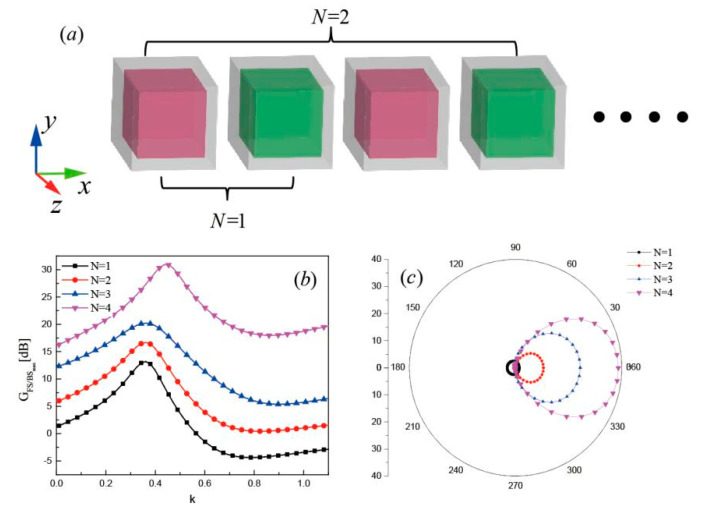
(**a**) Schematic of the core-shell nanocube dimer in a chain (**b**) Far-field forward-to-backward directionality $G_{FS/BS_{max}}$ and (**c**) Scattering patterns of the chained core-shell nanocube dimer for N = 1, 2, 3, and 4.

To improve the directionality, we show in Figure 7a the core-shell nanocube dimer with a linear chain along the *x* axis. The spectral dependence of the directionality on the number of core-shell nanocube dimers N (2× dimers) in the chain is observed from Figure 7b. As N was varied from 1 to 4, the peak position of $G_{FS/BS_{max}}$ red-shifts and the peak increased in height to 31 dB at k=0.45 for N = 4, compared to 13.2 dB for the dimer (N = 2). It can be inferred that a larger number in the nanocube dimer chain changes the scattering pattern of the nanoantenna. The scattering pattern of the chained core-shell nanocube dimer with N = 1, 2, 3, and 4 were numerically calculated and are presented in Figure 7c. Interestingly, the chained core-shell nanocube dimer performs as a lens that concentrates the radiation into a narrow beam and therefore this makes an efficient unidirectional nanoantenna.

Apart from plane wave excitation, the results from the electric dipole excitation of the core-shell nanocube dimer can be used as a rule of thumb to gauge unidirectional scattering of the nanoantenna. In this regard, not only is the directionality important, but also the Purcell factor is essential to the accurate interpretation for some fundamental scattering properties. To determine the modification effect of dipole emission, we calculated the Purcell Factor (PF) defined as the emitted power normalized to the radiated power [40,41,42]:

(9) $$PF=\frac{\gamma }{\gamma_{0}}=\frac{P}{P_{0}}$$

(10) $$P_{0}=\frac{\omega^{4}{\left|P_{0}\right|}^{2}}{12\pi \varepsilon_{0}c^{3}}$$

where P and $P_{0}$ are the power losses by the electric dipole with and without the resonator in vacuum.

**Figure 8:**
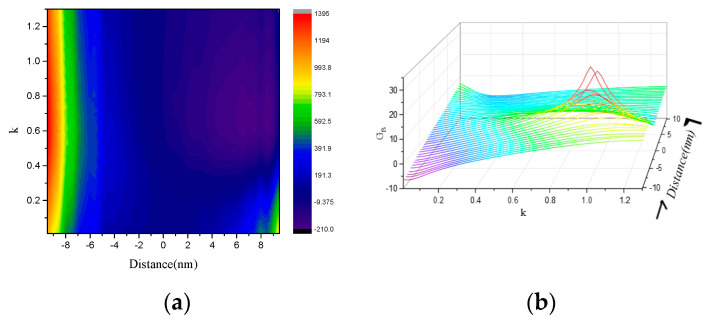
Electric dipole excitation: (**a**) Purcell factor and (**b**) Far-field forward-to-backward directionality $G_{FS/BS_{max}}$ versus the coefficient *κ* for different distances.

As shown in Figure 8a, the *x*-axis oriented electric dipole emitter was axially displaced from the center of the core-shell nanocube dimer by a distance *d* and the peak of the PF spectrum shifted to a shorter wavelength when the distance *d* was increased. The PF of the electric dipole almost reached 1400 at the coefficient *κ* = 0.644 in the core for *d* = −9.5 nm. Therefore, the distance between the electric dipole and nanoantenna is an effective parameter to manipulate the enhancement in the electric dipole (ED) emission. In order to gain further insight into the directionality, we show in Figure 8b the 3D plot of the far-field forward-to-backward directionality $G_{FS/BS_{max}}$ as a function of the coefficient *κ* for different distances. $G_{FS/BS_{max}}$ increased as the distance from the center of the nanoantenna was reduced from *d* = −10 to *d* = 0. Then $G_{FS/BS_{max}}$ decreased as the distance from the center of the nanoantenna was increased from *d* = 0 to *d* = +10. $G_{FS/BS_{max}}$ at *κ* = 0.9 reached 35 dB for *d* = 0. The results clearly demonstrate that the optical nanoantenna introduces remarkable emission enhancement in addition to tunable radiation directionality.

## 4. Conclusions

Highly switchable directionality was observed from the optical nanoantenna comprising a core-shell nanocube dimer with balanced gain and loss. By adopting the FEM and analytic expressions, the scattering behavior of the nanoantenna was found to depend on the phase difference of the emitting electric dipoles, and superior forward and backward radiation patterns can be achieved from the proposed optical nanoantenna by varying the coefficient *κ* in the core. Moreover, the far-field forward-to-backward directionality depends on the frequency, dielectric core, and emitting electric dipole position. The directionality of light scattering can be enhanced by arranging the core-shell nanocube dimer in a chain. The combination of nanostructures with balanced active and passive elements studied here can be utilized as one of the basic building blocks to construct metamaterials with directional optical responses. Additionally, the presence of the active and passive elements provides the possibility of being tunable through the control of the coefficient *κ* and enhancing the overall optical response due to the decrease in the inherent material losses of the system. The Au nanocube can be synthesized by means of a simple chemical approach. The dielectric core with balanced gain and loss can be loaded into the truncated nanocubes [43]. In general, the coefficient κ is relevant to the emission cross section σ_e_ and the concentration N in these systems, and the corresponding coefficient can be written as *κ*_core_ $=\frac{\lambda }{4\pi }N\sigma_{e}$ [10]. In practice, Er-doped silicon nanocrystals have achieved σ_e_ = 10^−16^ cm^2^, the corresponding coefficient can be estimated at a wavelength of 1550 nm as $N\sigma_{e}=3.0\times 10^{4}/\mathrm{cm}$ and κ_core_ = 0.37 [44]. Moreover, the concentration of dye molecules when casting T5oCx films by spin coating a chloroform solution on glass slides is 4.22 × 10^19^/cm^3^ and σ_e_ = 6.0 × 10^−16^ cm^2^ from pump-probe measurements of 610 nm, and κ_core_= 0.123 [45]. In this way, we can outline a scheme to provide objects which can be patterned or positioned on the nanoscale with these remarkable inverse gain/loss properties. The possibility of switching between forward and backward unidirectional scattering by varying the coefficient *κ* in the core of optical nanoantennas provides a powerful system for the focusing and manipulation of light on the nanoscale.

## Supplementary Materials

The following are available online at https://www.mdpi.com/2079-4991/10/8/1440/s1, Figure S1: Far-field forward-to-backward directionality $G_{FS/BS_{max}}$ with different (**a**) metal edge lengths *d*, (**b**) dielectric edge lengths *l*, and (**c**) face-to-face distance *a*.
