# Supplementary material for: Forward and Backward Unidirectional Scattering by the Core-Shell Nanocube Dimer with Balanced Gain and Loss
Source: Nanomaterials (Basel). 2020 Jul 23;10(8):1440. doi: 10.3390/nano10081440 (PMC7466381; doi:10.3390/nano10081440)
Supplement: Supplementary file 1 [file nanomaterials-10-01440-s001.pdf]

Supplementary Information

To clarify the influence of the geometry on the far-field scattering patterns for the coefficient *k* in the core, a systematic investigation was performed by varying three parameters *d*, *l*, *a*. As shown in Figure S1a and b, the curve exhibits pronounced directionality towards the opposite direction at the metal and dielectric edge lengths of *d*=50 nm and *l*=40 nm. The metal and dielectric edge lengths were fixed while the face-to-face distance *a* was varied. Figure S1c shows that the directionality $G_{FS/BS_{max}}$ increased as the face-to-face distance of the nanoantenna was increased from 5 nm to 20 nm. The directionality $G_{FS/BS_{max}}$ reached the maximum and a high directivity of scattering could be achieved when *d*, *l*, *a* were 50 nm, 40 nm, and 20 nm, respectively.

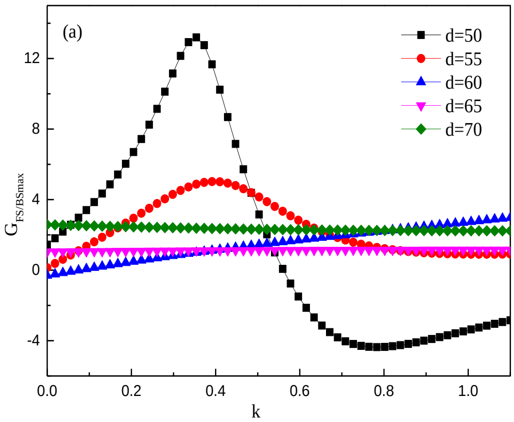

(**a**)

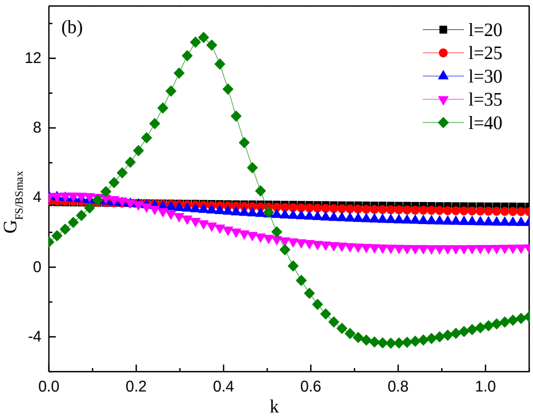

(**b**)

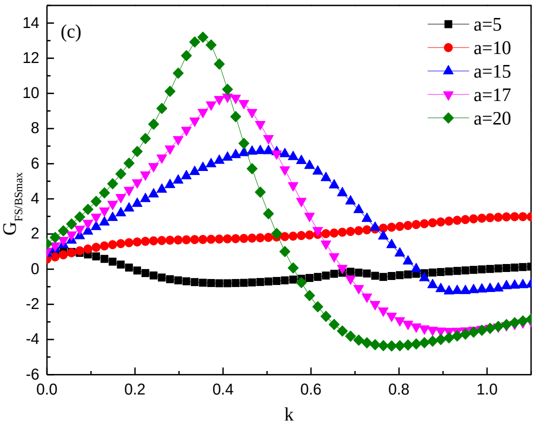

(**c**)

Figure S1. Far-field forward-to-backward directionality $G_{FS/BS_{max}}$ with different (**a**) metal edge lengths *d*, (**b**) dielectric edge lengths *l*, and (**c**) face-to-face distance *a*.
